# Nanofluidics for Pioneering Synthetic Biology of Bottom-Up Cell-Free Molecular Systems

**DOI:** 10.1021/acssynbio.6c00178

**Published:** 2026-06-12

**Authors:** Shiming Wu, Qun Ma, Madoka Takai, Yan Xu

**Affiliations:** † Department of Chemical Engineering, Graduate School of Engineering, 12936 Osaka Metropolitan University, Sakai, Osaka 599-8570, Japan; ‡ Department of Bioengineering, School of Engineering, The University of Tokyo, Bunkyo-ku, Tokyo 113-8656, Japan

## Abstract

Synthetic biology via bottom-up assembly is transitioning from stochastic, extract-based cell-free systems toward reconstituted, molecularly defined cell-free molecular systems. Transitioning to molecularly defined systems provides a path to quantitative design; however, the active assembly of these molecular building blocks into ordered spatiotemporal architectures remains a formidable challenge in synthetic biology. In this perspective, we propose nanofluidics as a transformative platform to bridge this gap. By leveraging nanoconfinement effects and precision mass transport, nanofluidics facilitates the active assembly of molecular building blocks into functionally integrated spatiotemporal structures, thereby pioneering the synthetic biology of bottom-up cell-free molecular systems. Specifically, we discuss how nanofluidics enables precise control over fluid dynamics and single-molecule behavior within nanochannels and facilitates molecular active-assembly and tunable interactions of molecular components by engineering design of nanofluidic devices. Furthermore, we highlight key challenges and opportunities using nanofluidics to build next-generation cell-free molecular systems with single-molecule resolution. This perspective provides a strategic roadmap for the synthetic biology of bottom-up cell-free molecular systems.

## Graphical abstract

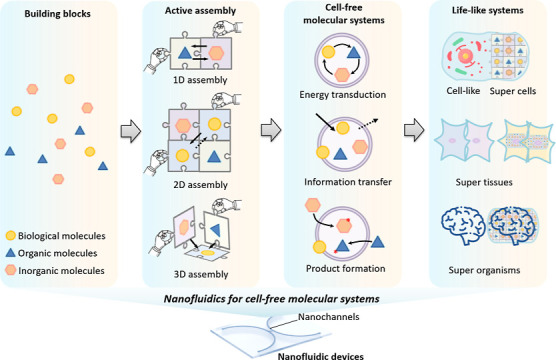

## Introduction

Synthetic biology aims to implement modular programming and functional reconfiguration of living systems through engineering design, offering transformative potential across diverse sectors, including life sciences, biopharmaceuticals, environmental remediation, and advanced biomaterials.^1–5^ Currently, the field progresses along two primary strategies: the top-down approach, manipulating existing natural systems, and the bottom-up approach, assembling synthetic life-like systems from molecular building blocks.^6–10^

Recently, the bottom-up strategy has been emerging because it allows for the rational design of life-like systems from the ground up, effectively bypassing the inherent complexity of living cells to achieve unprecedented mechanistic clarity and engineering precision.^11–13^ Central to this pursuit is the bottom assembly of cell-free molecular systems (CFMS), which have emerged as a pivotal platform characterized by their open architecture, programmability, and accelerated design–build–test–learn (DBTL) cycles.^14–16^ By rebuilding biological complexity from core processes such as transcription, translation, and metabolism, bottom-up CFMS makes molecular-scale function design more transparent and modular, advancing synthetic biology toward higher resolution control and more precisely tuned outputs.^17–20^ Despite these advancements, bottom-up assembly of CFMS with hierarchical architectures, dynamic compartmentalization, and spatiotemporal coordination remains a formidable challenge. Most current systems still rely on the stochastic mixing of molecules in bulk solutions, depending solely on molecular self-assembly for functional building blocks.^21–23^ It lacks robust engineering toolsets for active assembly and precise organization of building blocks at the molecular scale. As a consequence, how to achieve intricate assembly and organization of both natural and non-natural molecules remains an open challenge.

Nanofluidics has emerged as a promising engineering solution. Nanofluidics studies and exploits the behavior of fluids confined within nanoscale structures.^24–26^ Leveraging unique spatial nanoconfinements, nanofluidics enables the manipulation of fluid dynamics and single-molecule transport within nanochannels where dimensions are comparable to or smaller than the Debye length.^27–31^ While various nanofluidic formats exist, chip-based nanofluidic devices (hereafter referred to as “nanofluidic devices”) have emerged as the premier platform for synthetic biology, owing to their exceptional mechanical rigidity, superior optical transparency for single-molecule imaging, and highly reproducible architectures.^32–35^ By coupling space and surface effects, nanofluidic devices have been used as a versatile toolkit for single-molecule manipulation, guided assembly, and the modulation of molecular interactions, and provide ideal platforms to coordinate single-molecule behavior and construct CFMS. Specifically, nanofluidic devices are compact, planar, and often transparent solid-state platforms with precisely defined nanochannels fabricated by advanced nanolithography, shifting molecular transport from bulk-dominated behavior to interface- and charge-governed regimes.^36–40^ By integrating surface chemical functionalization with multiphysical field effects, these platforms can facilitate the deterministic capture, localization, and sequential arrangement of individual biomolecules.^41,42^ Furthermore, the generation of much smaller compartments in the nanochannels supports facile and real-time observation, quantitative statistical analysis, and high-throughput screening at the single-molecule level, providing an essential infrastructure for massively parallelized cell-free reactors.^43–45^ By transcending the limitations of conventional homogeneous systems, these capabilities shift biological and chemical assembly from molecular self-organization toward hand-like, flexible molecular manual-assembly, thereby unlocking the potential to engineer highly complex bottom-up cell-free synthetic platforms. We define this hand-like, precisely controlled mode of molecular assembly as molecular active-assembly (or molecular manual-assembly). It allows the pinpoint insertion of new building blocks or the selective removal of preassembled modules, thereby enabling the creation of new functions and even functionalities beyond those of the original system.

In this perspective, we offer a comprehensive overview of recent advances in nanofluidics for synthetic biology, with a focus on enabling programmable artificial molecule systems through controlled compartmentalization and nanoscale transport, single-molecule transport pathways, regulated, molecular active-assembly processes of single molecules, and tunable single-molecule interaction networks (Figure 1). Looking ahead, we further discuss the key challenges and emerging opportunities for using nanofluidics to bottom-up CFMS and, more broadly, next-generation artificial life platforms in engineering biology. Importantly, the physical confinement parameters introduced by nanofluidics themselves can serve as designable variables, shifting the design logic of CFMS from selecting molecular components to engineering physical environments that govern molecular behavior and system-level functions. In this sense, nanofluidics is not merely a supporting tool, but represents a substantive expansion of the design principles underlying bottom-up synthetic biology.

**Figure 1:**
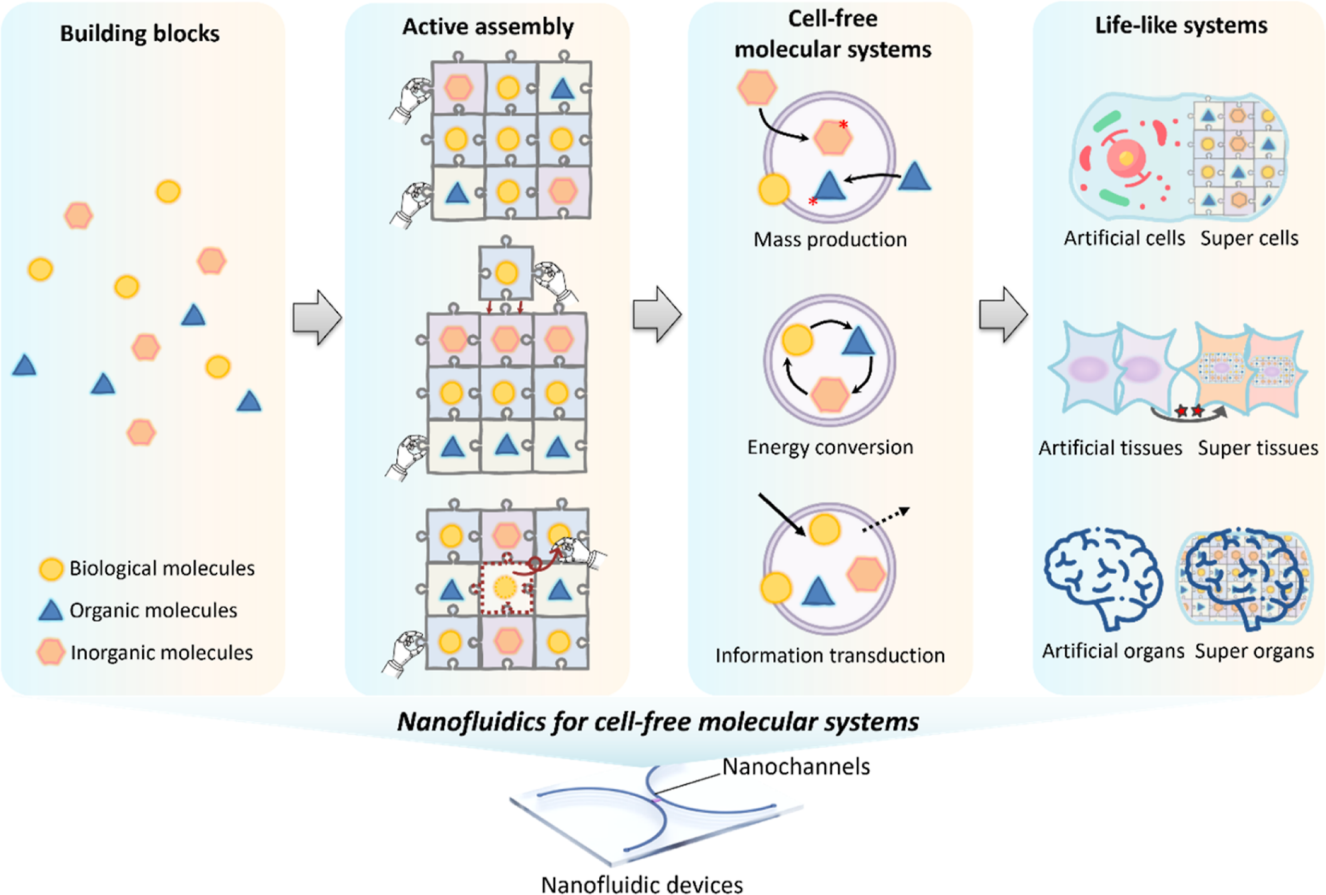
Nanofluidics for pioneering synthetic biology of bottom-up cell-free molecular systems. Nanofluidic devices offer a new route for bottom-up synthetic biology, enabling a transition from stochastic cell-free self-organization to compositionally defined, quantitatively designable CFMS. The schematic shows molecular active-assembly of biological, organic, and inorganic building blocks via nanoconfinement and precision transport to create spatiotemporally organized, compartmentalized architectures. This platform supports increasing complexity from synthetic cells to engineered tissues and organ-like systems.

### Nanofluidics-Based Molecular Active-Assembly of CFMS

Unlike traditional self-assembly processes that rely solely on intrinsic intermolecular interactions to drive spontaneous structural organization, the molecular active-assembly of CFMS holds great potential for externally guided, stepwise, and highly orchestrated construction of molecular building blocks. To actively assemble molecular building blocks into functional molecular systems, nanofluidic-based CFMS begins with the geometric engineering of nanochannels, where dimensions are tailored to exert precise and reproducible control over molecular behavior and conformations at the single-molecule level.

Over the past decades, nanofluidic devices have been emerging as a tool to investigate the unique transport phenomena of fluids and molecular carriers within nanoconfinements.^46–48^ The fabrication of these devices follows a typical top-down micronanofabrication procedure, characterized by multiscale patterning and precise bonding.^49^ As illustrated in Figure 2a, the core workflow begins with photolithography to define micrometer-scale inlet channels on a glass substrate. Subsequently, on a second glass substrate, electron-beam lithography is used to precisely pattern nanoscale features in designated functional areas, achieving sub-10 nm resolution. Dry etching then transfers these intricate patterns into the substrate with high fidelity. Finally, the two etched glass substrates undergo rigorous cleaning and are sealed via bonding, resulting in a robust, fully enclosed integrated chip that serves as a stable and transparent platform for the construction of CFMS.

**Figure 2:**
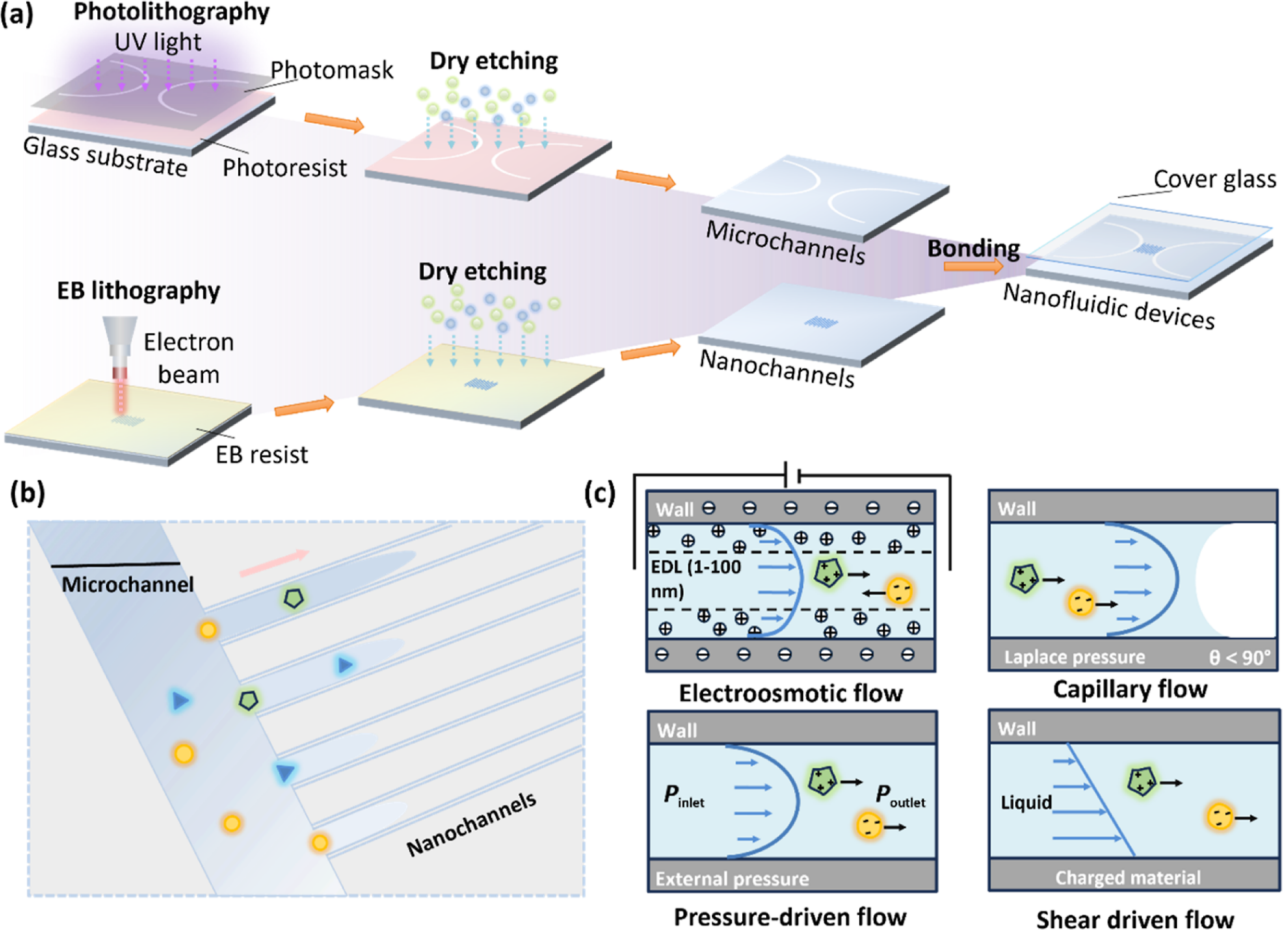
Fabrication and single-molecule assembly mechanisms of nanofluidic devices. (a) Schematic of the nanofluidic device fabrication workflow. (b) Schematic of the micro–nano fluidic interface. A microchannel delivers molecules or particles in solution and distributes them into a parallel nanochannel array. At the micro-to-nano transition, fluid moves from a low-resistance microchannel into high-resistance nanochannels, enabling controlled delivery into the nanochannels and nanoconfinement. (c) Representative mechanisms for regulating molecular and ionic transport within nanochannels.

Constructing nanofluidic-based CFMS lies in achieving precise interface engineering and active assembly of molecular building blocks. Technically, nanofluidic devices facilitate a seamless transition from macroscopic loading to single-molecule manipulation by integrating heterogeneous interfaces between micro- and nanochannels (Figure 2b). The primary transport mechanisms include electroosmotic flow (EOF) governed by electrical double layer overlap, capillary-driven flow (CDF) based on liquid–solid interfacial tension, pressure-driven flow (PDF) regulated via backpressure at the micro-nano interface, and high-stress shear-driven flow (SDF) (Figure 2c).^50–52^

Leveraging confined volumes ranging from pL to aL, these platforms can isolate individual molecular components within defined nanochannels according to statistical control. At the nanoconfinements, the translational and rotational degrees of freedom of molecules become strongly restricted, leading to orders-of-magnitude differences between the parallel and perpendicular diffusion coefficients, and consequently enhancing the anisotropy of molecular fluctuations. The diffusion coefficient of molecules in a 10 nm channel can decrease by one to 2 orders of magnitude compared with their bulk values. In addition, the folding energy barrier of a single molecule can be reduced by approximately 2–5 *k*_B_T in the vicinity of a nanoscopic interface. In confinement-induced ordering, polymer segments exhibit enhanced conformational order when restricted within narrow nanoscale spaces. Ultrasmall confined volumes can markedly increase the effective local concentration of reactants, thereby enhancing the reaction rate according to mass-action kinetics. Moreover, nanoconfinements and interfacial interactions can regulate molecular residence time, binding geometry, diffusion coefficients, and activation or folding energy barriers. These effects may alter key kinetic parameters, including the association rate constant (*k*_on_), dissociation rate constant (*k*_off_), catalytic turnover number (*k*_cat_), Michaelis constant (*K*_m_), and apparent activation energy (*E*_a_), thereby quantitatively modulating association/dissociation kinetics, conformational transitions, and catalytic turnover.

Furthermore, compared with molecular self-assembly, the molecular active-assembly of CFMS not only emphasizes the order and programmability of the construction process, but also allows for the deliberate design of the interaction-defined “assembly order” between molecular building blocks. By precisely coupling physical fields with surface charges, these mechanisms enable deterministic control over molecular assembly, establishing the physical foundation for constructing life-like systems with spatiotemporal coordination.

### Interface Engineering-Enabled Reconfiguration of Compartmentalized Functions

Compartmentalization is fundamental to biological function. Interfaces formed by cellular and organellar membranes subdivide biochemical reaction space into distinct microcompartments, thereby supporting parallelization of incompatible reactions, preservation of concentration gradients, and spatially localized signal amplification.^53,54^ However, it limits and constrains the precise reconstitution of biochemical networks within artificial compartments.

Nanofluidics, empowered by interfacial engineering, provides a dual-mode “static–dynamic” regulation paradigm to address this bottleneck and to recapitulate compartmentalization-enabled functions in living systems (Figure 3). In the static regime, studies have demonstrated that stable gas–liquid interfaces can be engineered and pinned within nanochannels via hydrophilic/hydrophobic nanopatterning, providing three compartmentalization functions.^55^ First, it enriches target molecules to create a locally high-concentration microdomain. Second, it suppresses cross-interface transport by diffusion, because the physicochemical disparity between the liquid and gas phases strongly limits molecular permeation. Third, it partitions the microenvironment, allowing distinct solvation conditions to be maintained on either side of the interface. In the dynamic regime, studies have shown that temperature can be used to control valve opening and closing, providing a feasible route to direct molecular transport between neighboring artificial compartments and to regulate reaction order during biochemical processes.^56^ This “dynamic gating” behavior is conceptually aligned with regulated intercompartment exchange in cells, such as that mediated by the nuclear pore complex and mitochondrial membrane transporters.

**Figure 3:**
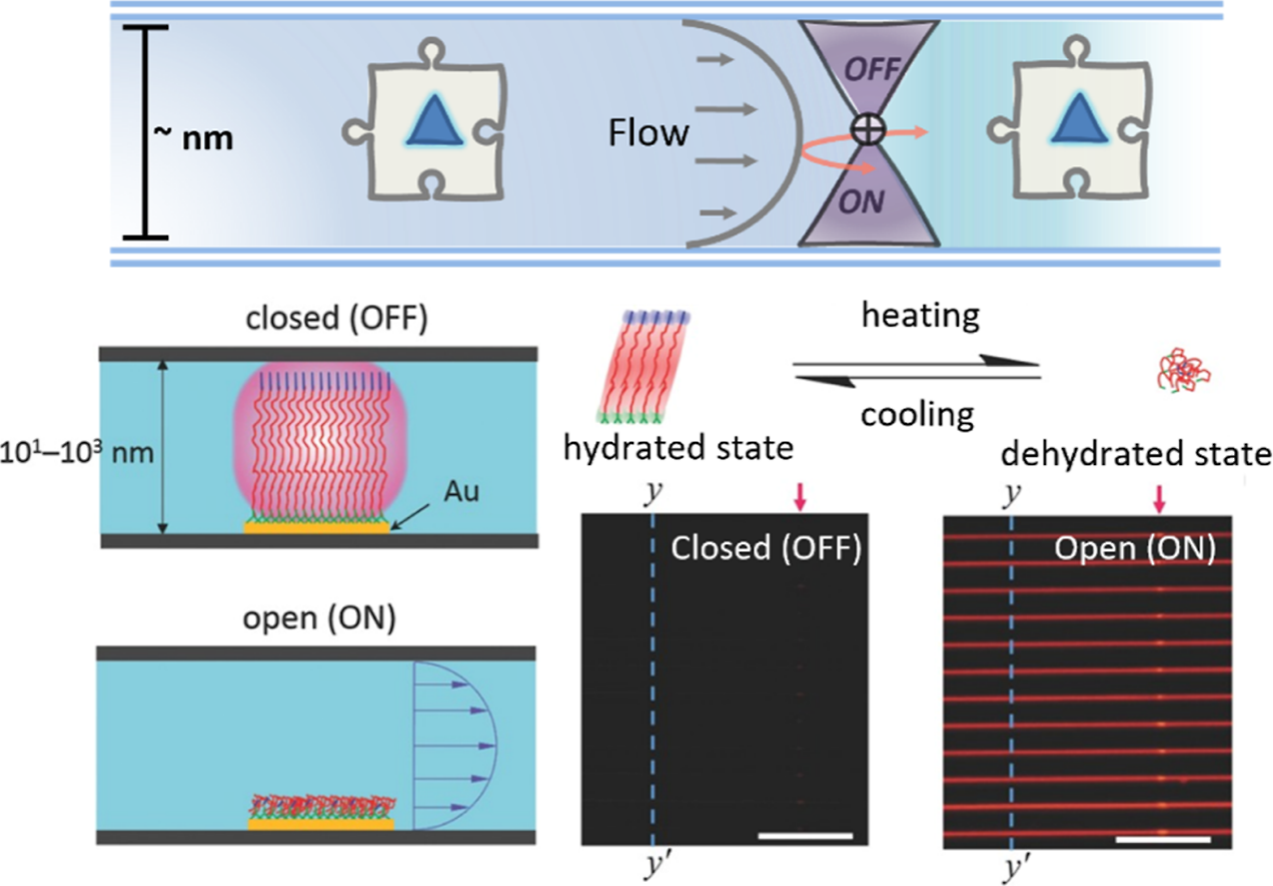
Interface engineering-enabled reconfiguration of compartmentalized functions. Top: concept of switchable nanovalves integrated into a nanochannel regulates molecular passage/blocking (ON/OFF) and modulates flow pathways. Bottom left: a stimulus-responsive soft layer swells in the hydrated state to close the channel (OFF) and shrinks upon dehydration to reopen it (ON). Bottom right: experimental characterization of fluorescence images showed the transition between OFF and ON states, enabling programmable compartmentalization. Reproduced with permission from ref 56. Copyright 2016 WILEY-VCH Verlag GmbH & Co. KGaA, Weinheim.

### Construction and Regulation of Single-Molecule Transport Pathways

Ensuring material transport and signal transduction at the single-molecule level constitutes a fundamental challenge in the construction of CFMS. Material exchange and signal transduction in biological systems are orchestrated by cytoskeleton-guided transport and the selective barrier of the nuclear pore complex.^57,58^ These mechanisms leverage confinement from nanometre-scale pores to subcellular corridors to restrict diffusion and tune molecular encounter rates, thereby shaping assembly efficiency and product specificity. Researchers have used nanofluidics to achieve precision single-molecule transport pathways.^59^ By integrating a flexible thin-glass layer into the nanofluidic device, external pressure can be applied to reversibly gate the nanochannels, enabling precise blocking and release of individual small-molecule flows (Figure 4). This platform enables the simulation of spatial cascade assembly of enzyme complexes in natural metabolic pathways, facilitating efficient mass transport, production, and signal transduction.

**Figure 4:**
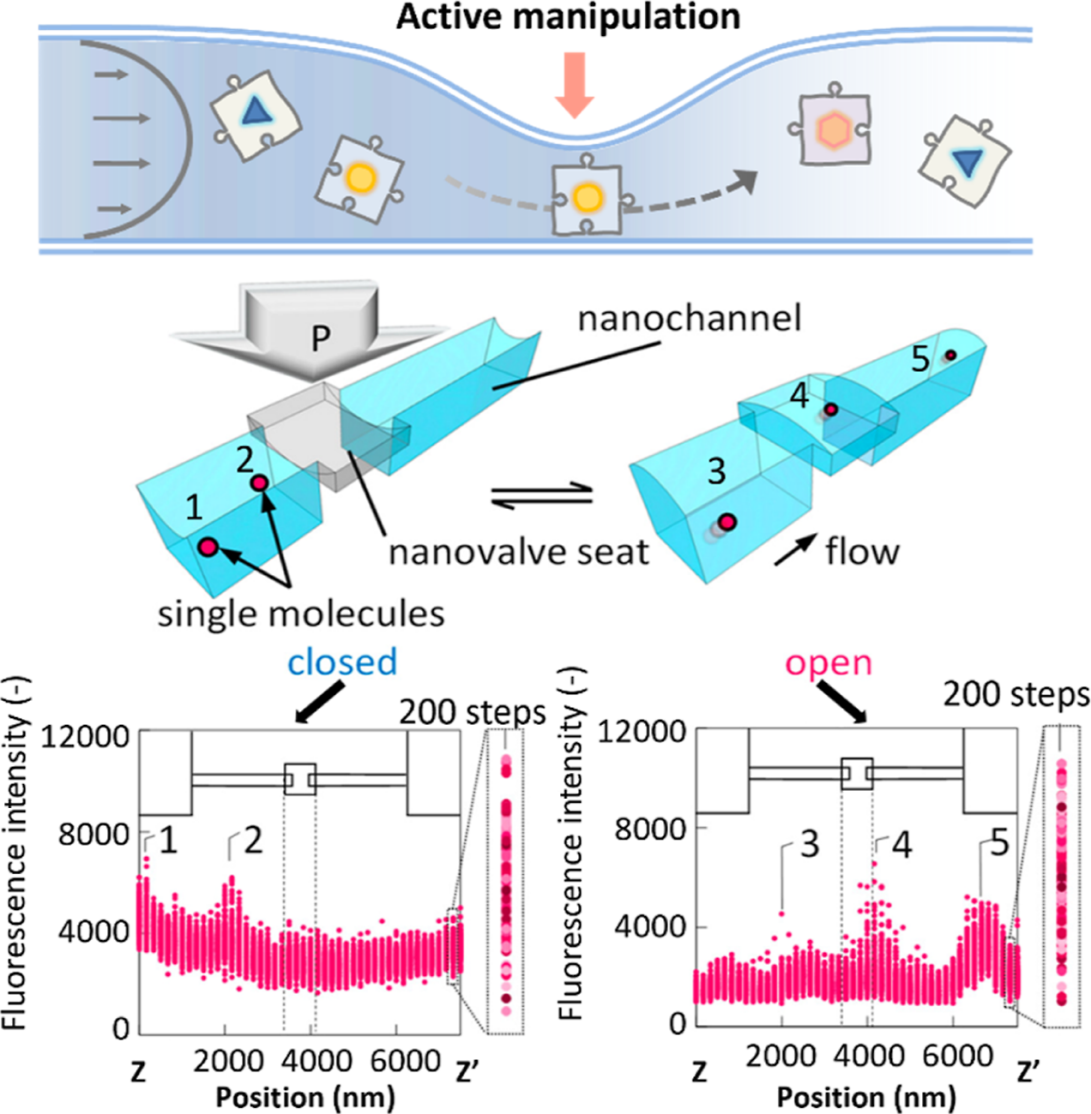
Construction and regulation of single-molecule transport pathways. A flexible thin-glass layer enables pressure-actuated nanovalves that reversibly switch nanochannels between closed and open states. This gating provides on-demand blocking, release, and directional transport of single small molecules, supporting controlled mass transport and signal propagation. It can also mimic spatially cascaded assembly along metabolic pathways to enhance transport efficiency and product formation. Reproduced from ref 59 with permission.

### Regulated Molecular Active-Assembly Processes of Single Molecules at Physiological Concentrations

A key challenge in bottom-up CFMS is how to assemble molecular building blocks and encode their positions at physiological concentrations (nM−μM).^60^ Conventional single-molecule assays typically dilute samples to the pM range to avoid multiple occupancy, which moves the system far from natural cellular conditions. Microarray platforms can capture molecules, but micrometre-scale features typically load hundreds to thousands per capture spot, preventing single-molecule readout.

Recently, researchers developed a nanofluidic aptamer nanoarray for single-molecule active-assembly and tunable single-molecule interaction networks. Using a nano-in-nano integration strategy,^61^ the platform constructs high-density nanopatterned arrays inside nanochannels and enables site-specific self-assembly of single proteins, providing nanometre-scale spatial addressability. This design avoids the pM-level dilution typically required for conventional single-molecule assays, allowing spatial encoding and precise localization of individual biomolecular building blocks at near-native concentrations. It also enables interrogation of functional heterogeneity at the single-molecule level (Figure 5). By locally enriching target molecules within ultrasmall confined volumes while spatially separating capture events, this platform can maintain effective reaction probability and reduce signal overlap and nonspecific interference, thereby helping preserve reaction efficiency, robustness, and single-molecule precision at near-physiological concentrations. This work provides key insights into molecular active-assembly of molecular building blocks at near-physiological concentrations and ultrasmall volumes into CFMS.

**Figure 5:**
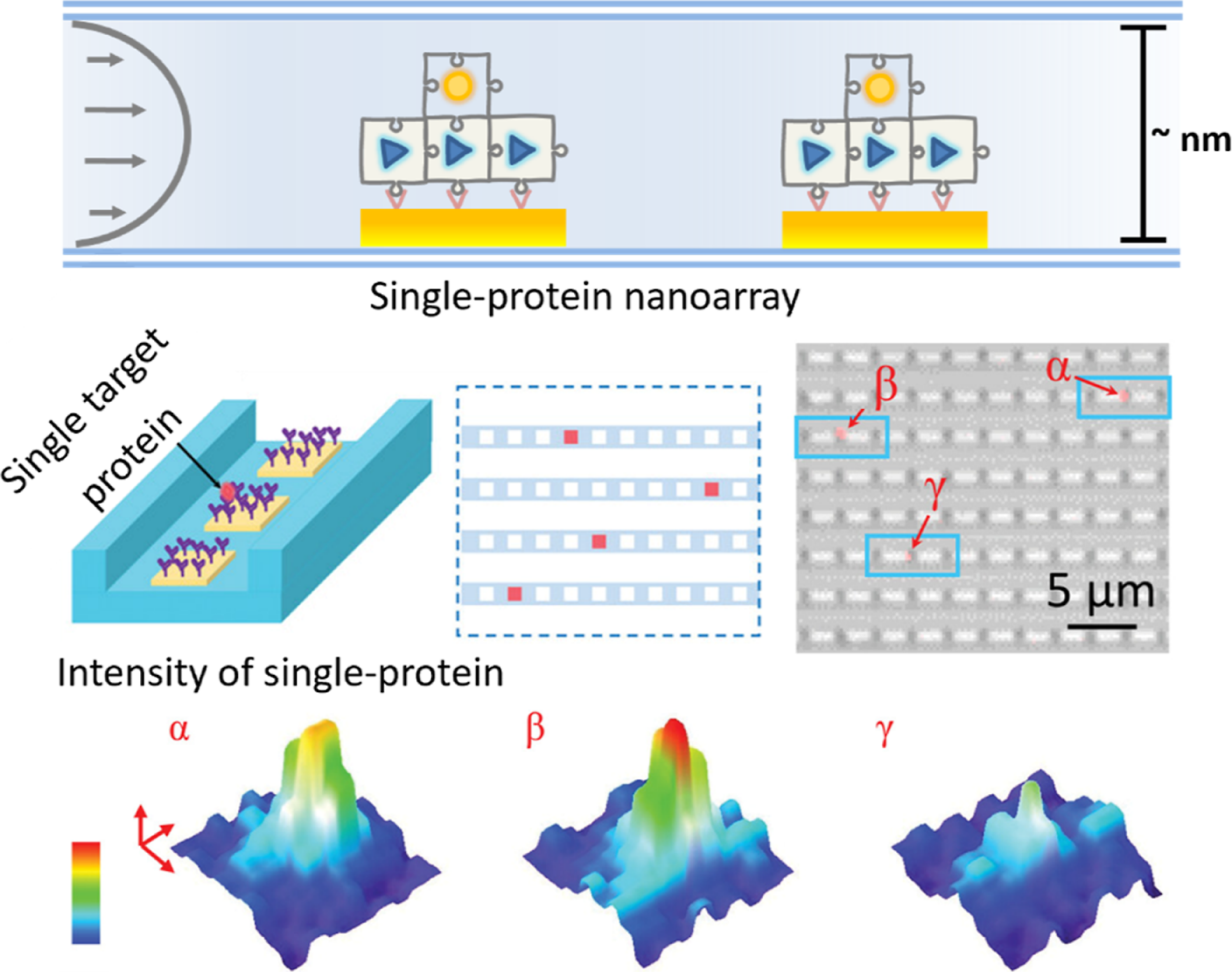
Regulated molecular active-assembly processes of single molecules at physiological concentrations. Under nanofluidic confinement, single target proteins can be stochastically captured at near-physiological concentrations, with their positions spatially encoded for addressable readout. Top: molecules flow through nanochannels and are enriched at predefined capture sites. Middle: surface-functionalized nanostructures form high-density capture arrays; representative events are labeled (α, β, γ). Bottom: 3D signal heatmaps show localized, reproducible single-protein signatures across sites. Reproduced with permission from ref 61. Copyright 2023 The Authors. Published by Wiley-VCH GmbH.

## Challenges and Outlook

The ultimate goal of synthetic biology is to construct programmable artificial life systems through precise active-assembly of molecular building blocks. The core framework of synthetic biology is the DBTL cycle (design–build–test–learn).^62^ In conventional DBTL workflows, it often has to rely on complex intracellular networks or self-organization in bulk solutions, making it difficult to precisely control the spatial positioning of individual molecules and the timing of reactions.^63,64^ Despite recent progress, nanofluidic-based CFMS designed for future synthetic biology still faces several key challenges, including achieving high spatiotemporal precision, controlling single-molecule reaction pathways with high stability and selectivity, maintaining reliable information-transfer fidelity, and improving the yield per operation during molecular construction (Figure 6a).

**Figure 6:**
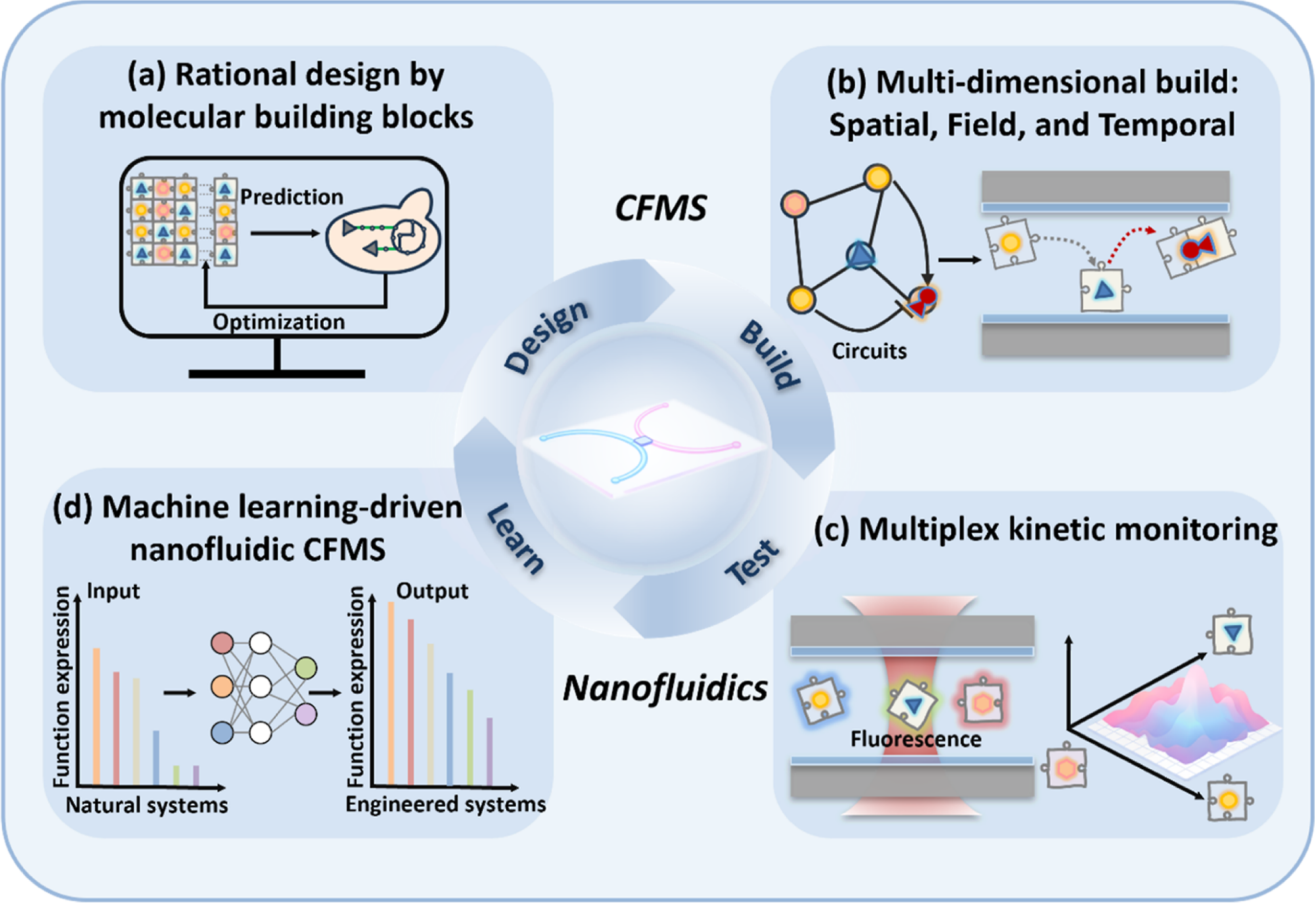
Nanofluidics-based CFMS for future synthetic biology and engineering biology. (a) Design: candidate circuits or systems are computationally designed through the prediction and optimization of functional elements, regulatory modules, and their combinatorial relationships. (b) Build: nanofluidic devices enable precise circuit construction through spatial control (molecular localization and compartmentalization), field-mediated regulation (external physical or chemical cues), and temporal control (dynamic sequential assembly), thereby allowing the controlled integration and functional coordination of CFMS modules. (c) Test: by integrating nanofluidic devices with single-molecule fluorescence detection, the kinetics of biochemical reactions in CFMS can be monitored in a quantitative, multiparametric, and high-throughput manner. (d) Learn: deep learning models integrate input–output data from natural and engineered systems to uncover molecular design rules and guide functional optimization in engineered systems, forming a data-driven closed-loop framework.

### Spatiotemporally Ordered Molecular Active-Assembly of Building Blocks

A hallmark of living systems is that highly ordered functional organization does not arise by eliminating molecular randomness. A central challenge for bottom-up CFMS is therefore to recreate this “ordered randomness”. The goal is neither to fully suppress Brownian motion (physically impossible) nor to allow uncontrolled diffusion to dissolve function, but to program the physical microenvironment so that assembly becomes spatiotemporally controllable against a background of stochastic single-molecule motion. Whereas spontaneous molecular self-assembly follows the free-energy landscape and is constrained by probabilistic kinetics, molecular active-assembly using nanofluidic devices enables external, programmable modulation of energy barriers and interaction potentials, thereby achieving a level of pathway control and structural precision unattainable by self-assembly alone. Leveraging nanofluidic devices to achieve spatiotemporally ordered molecular active-assembly of single-molecule building blocks, thereby opening avenues for highly efficient material production and information transduction in synthetic biological systems (Figure 6b).

### Stability and Selectivity of Molecular Building Blocks

Natural biomacromolecules placed in nanoconfined environments are exposed to a combination of strong interfacial effects, geometric constraints, and coupled multiphysics perturbations. As a result, their conformational stability, interaction landscapes, and reaction kinetics can deviate substantially from behavior in bulk solutions. It will require strategies such as precisely tunable surface chemistries, soft-matter coatings, and designs that emulate cytoplasmic crowding to create “molecule-friendly” nanoreactors that both preserve native conformations and enable new functions (Figure 6c). Furthermore, efficient material production, information transduction, and energy transfer fundamentally rely on the specificity, selectivity, and stability of the underlying molecular interactions and reaction events, underscoring the need for assembly strategies that maintain precise control at the molecular scale. Finally, the development of integrated analytical approaches capable of real-time and multiplex kinetic monitoring of material, information, and energy flows during molecular assembly remains an essential challenge.

### From Molecular Active-Assembly to System-Level Functional Emergence

Despite the achievements, it still needs to ensure that assembled molecular components yield well-defined chemical, biological, or system-level functions (Figure 6d). For example, even if an enzyme cascade is arranged in a prescribed topology inside a nanochannel, how can one guarantee that cooperative catalysis surpasses bulk performance? Addressing this requires a “molecular engineering” mindset: viewing nanofluidic devices as programmable “molecular operating systems”. By coordinating interfacial engineering, geometric constraints, and molecular recognition, one can control local reactant concentrations, diffusion pathways, and interaction timing with single-molecule precision—so that the assembly process itself becomes a driver of functional emergence.

### Integration and Scaling for Engineering Biology

How to transition laboratory-scale demonstrations into robust, scalable, and engineering-grade applications remains an enduring and critical challenge. Breakthroughs will require coordinated progress in three directions: (1) Standardization: establish “universal interface” standards for nanofluidic devices (e.g., channel geometry specifications and surface-chemistry protocols) so that functional modules, interfacial templates, geometric encoders, and molecular-recognition arrays can be plugged in and used interchangeably. In addition, standardization of nanofluidic platforms should extend beyond device fabrication to include quantitative benchmarks for transport and reaction processes, such as ionic conductance, selectivity, residence time, and kinetic rate constants. Establishing such metrics, together with DBTL-compatible data structures, would enable systematic comparison, model-guided optimization, and scalable integration with synthetic biology systems. (2) Automation and machine learning-driven nanofluidic-based CFMS: develop nanofluidic DBTL closed-loop platforms compatible with biofoundry workflows, integrating AI-driven active learning to automatically extract design rules from single-molecule trajectories, transport dynamics, kinetic fingerprints, and structure–function relationships, and then feed the extracted rules back to guide the next round of nanochannel design, surface engineering, molecular combination, and operating conditions. This deep integration would enable nanofluidic devices to function not only as testing platforms, but also as programmable and self-optimizing engines for synthetic biology DBTL cycles; (3) Scale-up: adopt parallelization concepts from the semiconductor industry to build high-density nanofluidic arrays (e.g., chips with millions of nanochannels), transforming single-molecule experiments from “rare-event observation” into “high-throughput statistical analysis”, and generating the massive data sets needed for data-driven synthetic biology.

Finally, nanofluidic-based CFMS could move synthetic biology from genetic programming toward programming spatial organization at the single-molecule level, allowing for an engineered reconstruction of the fundamental organizational logic of life. By integrating engineering principles with automated methodologies, nanofluidic-based CFMS would offer insights for the predictable, programmable design and scalable manufacturing of biological systems; this paradigm further catalyzes the evolution of engineering biology, which leverages synthetic biology as its core foundational technology.
